# Machine learning model for predicting epidermal growth factor receptor expression status in breast cancer using ultrasound radiomics

**DOI:** 10.3389/fonc.2025.1683164

**Published:** 2025-10-17

**Authors:** Zhirong Xu, Jiayi Ye, Huohu Zhong, Jiemin Chen, Han Wang, Xiaoqian Zhang, Guorong Lyu, Shanshan Su

**Affiliations:** ^1^ Department of Ultrasound, The Second Affiliated Hospital of Fujian Medical University, Quanzhou, China; ^2^ Department of Nuclear Medicine, The Second Affiliated Hospital of Fujian Medical University, Quanzhou, China

**Keywords:** breast cancer, machine learning, epidermal growth factor receptor, ultrasound, radiomics

## Abstract

**Background/objectives:**

The epidermal growth factor receptor (EGFR) is a clinically important target, as its expression in patients with breast cancer influences both overall and disease-free survival. Current methods for assessing EGFR expression status in a patient are invasive. Therefore, in this study, we developed a machine learning-based approach utilizing ultrasound radiomics to non-invasively predict EGFR expression status in patients with breast cancer.

**Methods:**

Radiomic features were extracted from grayscale and wavelet-transformed ultrasound images of 321 patients. The dataset was randomly split into training (n = 225) and test (n = 96) sets at a 7:3 ratio with stratified sampling to preserve the EGFR+/– ratio. Key predictors were identified using a multi-step procedure—including reproducibility filtering (ICC > 0.75), univariate F-test filtering (p < 0.05), and L1-regularized selection via LASSO regression. Seven machine-learning models were trained. Model interpretability was assessed using SHAP (Shapley Additive Explanations). In addition to the hold-out evaluation, we performed stratified 10-fold cross-validation to reduce selection bias.

**Results:**

The random forest model demonstrated the optimal performance, with an area under the receiver operating characteristic curve of 0.86 in the training set and 0.70 in the test set. It significantly outperformed the other models (P < 0.001). The Shapley additive explanation method was used to interpret the model, revealing that original_ngtdm_Coarseness, original_ngtdm_Strength, and wavelet.LL_glcm_ClusterProminence were the top predictors. These features reflect structural compactness and heterogeneity associated with EGFR overexpression.

**Conclusions:**

We present a reliable and interpretable tool for non-invasively assessing EGFR expression status in patients with breast cancer. The most important predictors captured tumor heterogeneity and microstructural uniformity, highlighting the biological relevance of radiomic patterns in EGFR-positive tumors. This model integrates advanced imaging analyses with machine learning, underscoring the potential of radiomics to advance precision oncology.

## 1 Introduction

Breast cancer is one of the most prevalent malignancies among women, with an estimated 357,200 new cases recorded annually in China, accounting for 57.4% of the global incidence (1, 2). Despite advancements in treatment modalities such as neoadjuvant chemotherapy, surgery, and adjuvant therapy (3–5), there is a critical need to refine diagnostic and therapeutic strategies to enhance patient outcomes. The epidermal growth factor receptor (EGFR) plays a pivotal role in cell proliferation and differentiation (6). Its overexpression significantly accelerates metastasis and recurrence, leading to a marked decrease in overall and disease-free survival. Therefore, the EGFR is a clinically important therapeutic target that offers opportunities for innovative treatment strategies. However, the detection of EGFR overexpression in breast cancer primarily relies on invasive procedures, which can increase patient discomfort, procedural risks, and overall testing costs and complexity (7, 8). Consequently, there is an urgent need to develop a non-invasive and efficient method for predicting the risk of EGFR mutations in patients with breast cancer before treatment. Such an approach could shorten diagnostic timelines and reduce reliance on invasive procedures, providing essential guidance for personalized treatment planning.

Li et al. analyzed ultrasound images of 62 patients with breast cancer that were interpreted by experienced sonographers and found that lateral shadows and microlobulated margins were significantly associated with high cytokeratin 5/6 and EGFR expression (9). However, with conventional ultrasound techniques, it is challenging to differentiate between basal-like and normal-like breast cancer subtypes. Recently, the integration of artificial intelligence in clinical medicine has led to increased interest in radiomics, which autonomously extracts imaging features, quantifies tumor heterogeneity, and characterizes biological properties through high-throughput image analysis (10). Radiomics has also shown promise in predicting the genetic subtypes of breast cancer (8, 11–14). Machine learning (ML) enables computers to identify patterns and acquire knowledge by leveraging algorithms and mathematical principles, enabling continuous performance improvements (15–17). Compared to traditional statistical methods, ML techniques excel at uncovering hidden information within data, demonstrating superior learning and generalization capabilities (18). However, the limited interpretability of ML models represents a major challenge (19). The underlying mechanisms driving ML decisions can be difficult to discern, raising concerns about the reliability of the results. In medical diagnostics, interpretability is crucial because transparent models enhance the reliability and safety of decision-making outcomes. Only by ensuring model transparency can decision-making be deemed more reliable and safer (18). Currently, predictions of breast cancer genetic subtypes primarily focus on biomarkers such as estrogen receptor (ER), human epidermal growth factor receptor 2 (HER2), and cell proliferation index (Ki-67) (11–13). Although previous studies have explored imaging or genomic signatures for predicting EGFR expression in other cancers. To date, no studies have directly applied machine learning-based radiomics approaches on ultrasound imaging to predict EGFR expression status in breast cancer, highlighting a novel research gap addressed by this study (20, 21).

In this study, we developed and evaluated seven ML models—logistic regression (LR), support vector machine (SVM), k-nearest neighbors (KNN), random forest (RF), decision tree (DT), naive Bayes (NB), and neural network (NN)—to identify the optimal risk prediction model. Additionally, the Shapley additive explanation (SHAP) method was employed to quantify the contribution of each feature variable using both global and local interpretability approaches, thereby elucidating the key factors associated with predicting EGFR expression status in patients with breast cancer. In this study, we aimed to provide a non-invasive and accurate tool for assessing EGFR expression status. This tool could help optimize clinical management strategies and enhance patient quality of life.

## 2 Materials and methods

### 2.1 Patients and study design

**Figure 1:**
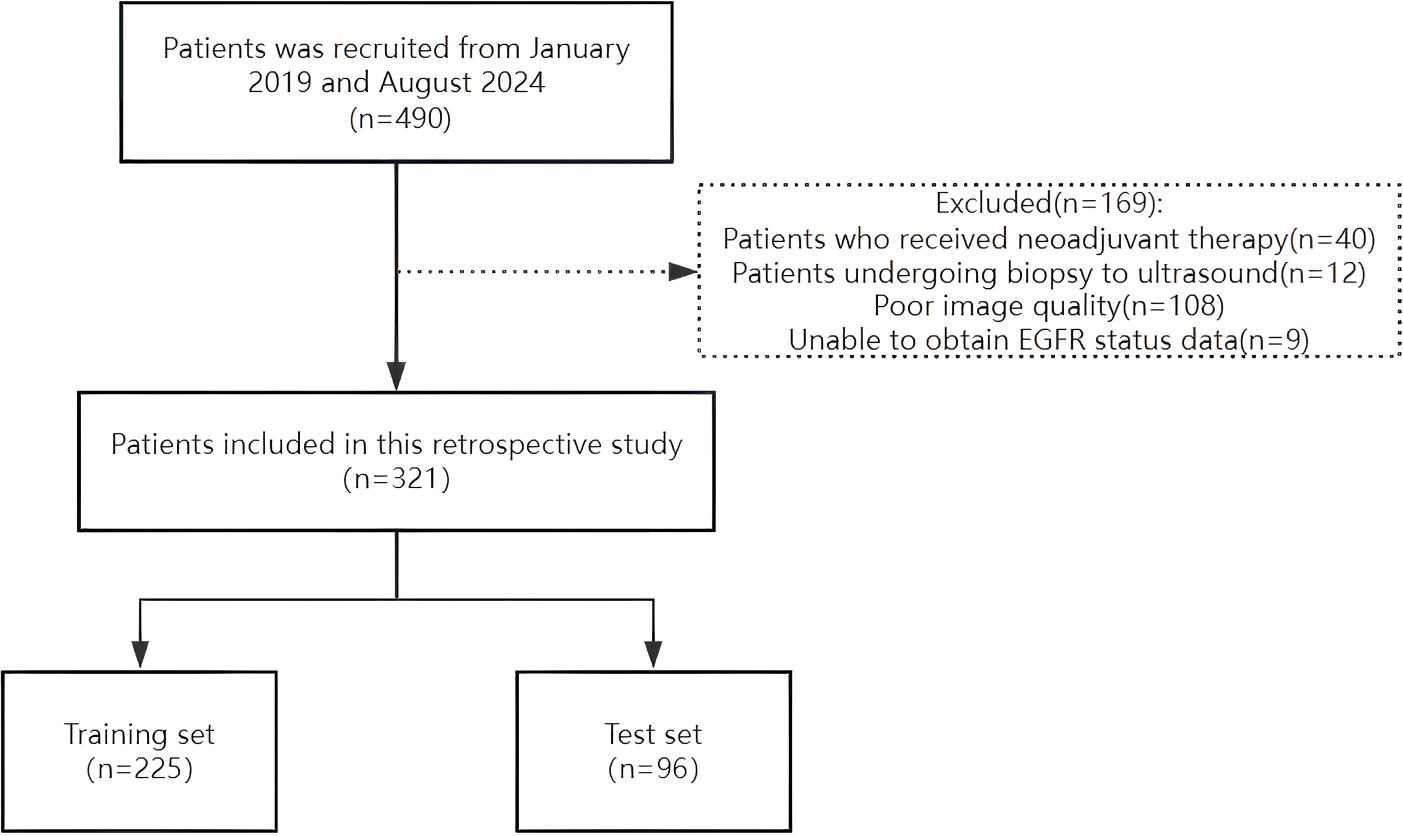
Flowchart of the patient recruitment process.

The Ethics Committee of the Second Affiliated Hospital of Fujian Medical University approved this study (Approval No. 021) on 26 March 2025, and all patients provided written informed consent. A retrospective analysis was conducted on female patients diagnosed with breast cancer through surgical pathology who underwent EGFR gene testing at our institution between January 2019 and August 2024. The inclusion criteria were 1) patients who underwent grayscale ultrasound and 2) those who underwent ultrasound examination within 2 weeks prior to genetic testing. The exclusion criteria were as follows: 1) patients who received neoadjuvant chemotherapy, 2) patients who underwent biopsy before the ultrasound examination, and 3) patients with unclear ultrasound images. A total of 321 grayscale ultrasound images from eligible patients were analyzed. These patients were randomly divided into training (n = 225) and test (n = 96) sets at a 7:3 ratio (**Figure 1**). To reduce selection bias beyond a single hold-out split, we additionally performed stratified 10-fold cross-validation, preserving the EGFR+/– ratio in each fold. Mean AUC, accuracy, precision, recall, and F1-score across folds were computed and reported. Clinical information of each patient was recorded, including age, maximum tumor diameter, tumor morphology, taller-than-wide orientation, presence of microcalcification, posterior acoustic attenuation, blood flow signals, and EGFR expression status. To preserve the class distribution (EGFR–:EGFR+ ≈ 2:1), all data partitions employed stratified sampling.

### 2.2 EGFR expression analysis

EGFR expression was assessed using immunohistochemistry on formalin-fixed, paraffin-embedded surgical specimens. Tissue microarray cores were selected based on representative tumor areas. EGFR protein expression was evaluated using the EGFR pharmDx Kit, with scoring based on membranous staining: 0 (no or weak staining in <10% of cells), 1+ (weak staining in ≥10% of cells), 2+ (moderate staining in ≥10% of cells), and 3+ (strong staining in ≥10% of cells). Tumors were classified as EGFR-overexpressing (EGFR+) if they scored 1+, 2+, or 3+, and EGFR-negative (EGFR-) if they scored 0. These immunohistochemical results were used as the ground truth labels (EGFR+ vs. EGFR–) for model training.

### 2.3 ROI segmentation and feature extraction

All patients underwent an ultrasound examination prior to surgery. Gray-scale ultrasound images were used for radiomic feature extraction. The ultrasound images were retrieved from the Picture Archiving and Communication System and saved in their original Digital Imaging and Communications in Medicine format. An ultrasound diagnostician with 10 years of experience (Reader A), who was blinded to clinical information, treatment methods, clinical outcomes, and pathological data, manually delineated the regions of interest (ROI) of the tumors using 3D Slicer software (version 4.11, https://www.slicer.org/). The tumor was identified based on the largest cross-sectional plane for ROI delineation and feature extraction. Two weeks after the initial delineation, Reader A and another ultrasound diagnostician with 15 years of experience (Reader B) randomly selected 30 images for ROI delineation to evaluate both inter- and intra-observer reproducibility of ultrasound radiomic feature extraction. Radiomic features with an intraclass correlation coefficient (ICC) greater than 0.75 were considered highly reliable and retained for model construction. Annotation information was removed from all images before delineation, and the results were saved in an ROI (nrrd) format. High-order texture features with low ICC (<0.75) were excluded due to their sensitivity to boundary placement, indicating poor inter-observer reproducibility. Dice similarity between segmentations was not computed, as the focus was on feature-level reliability rather than spatial overlap.

### 2.4 Radiomic feature extraction and selection

Ultrasound radiomic features were extracted from the two-dimensional ROIs in each patient’s ultrasound images using the open-source Python package Pyradiomics (version 3.8.8). Radiomic features were extracted from the original images without wavelet or LoG filtering. A multi-step feature selection pipeline was implemented to reduce overfitting and improve model generalizability. The feature selection process in the training set involved the following steps: (i) retain features with ICC > 0.75 from inter- and intra-observer tests; (ii) apply z-score normalization to all features; (iii) perform a univariate F-test (p < 0.05) to identify features with significant group differences as a preliminary dimensionality-reduction step; and (iv) apply L1-regularized logistic regression (LASSO) with 10-fold internal cross-validation as the final selector. A significance threshold of p < 0.05 was used without Bonferroni correction, as the subsequent LASSO step provides further regularization.

As a sensitivity analysis, we performed an ablation that removed step (iii) and applied LASSO directly; performance remained comparable to the full pipeline (see **Supplementary Table S1**), indicating that conclusions do not hinge on the univariate pre-filter.

In total, 464 features were extracted from each image, including shape features, first-order statistics, gray-level co-occurrence matrix (GLCM) features, gray-level run-length matrix (GLRLM) features, gray-level size zone matrix (GLSZM) features, and neighborhood gray-tone difference matrix (NGTDM) features.

### 2.5 Model construction

**Figure 2:**
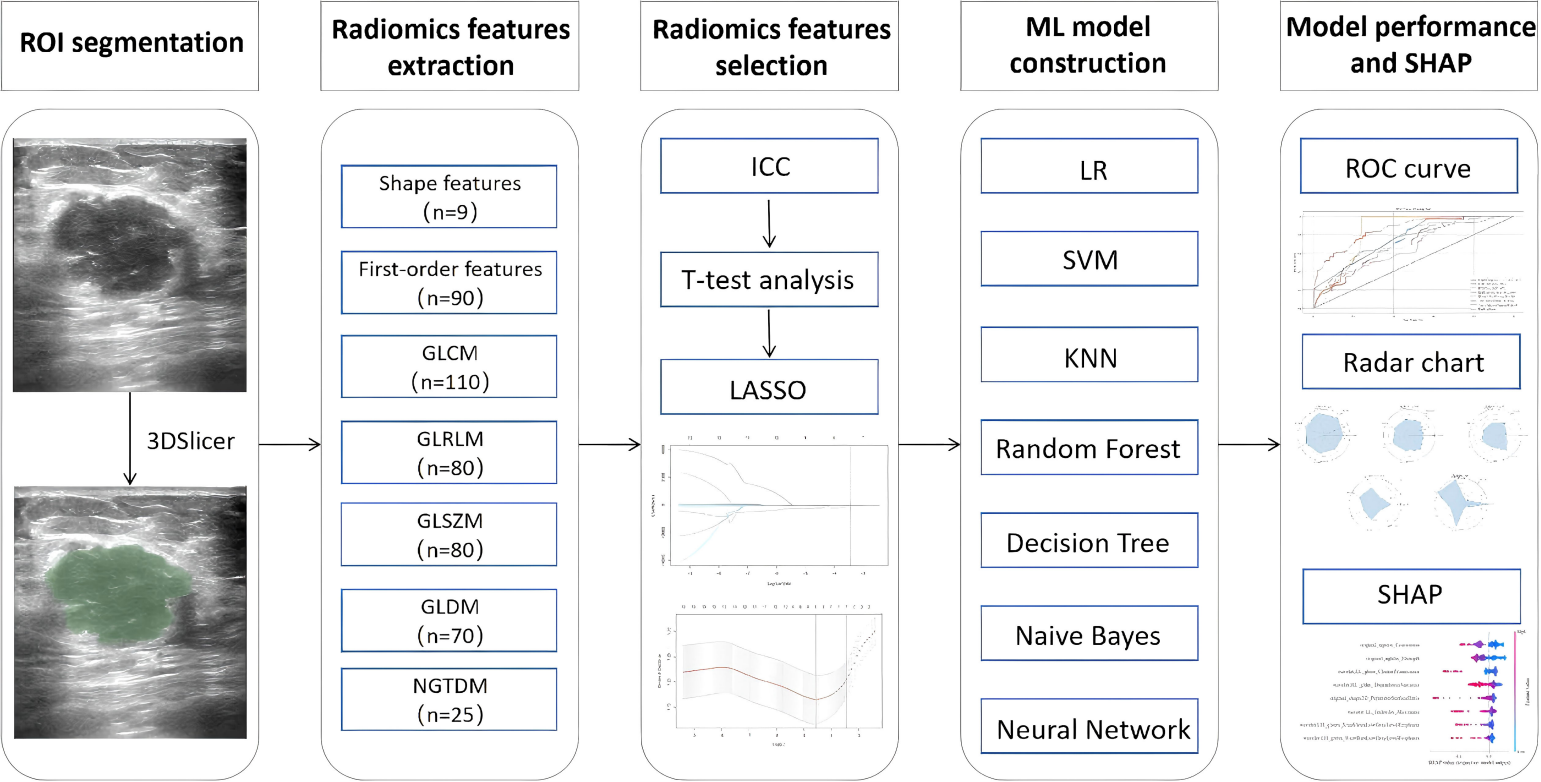
Overall workflow of the study. ROI, region of interest; ICC, inter- and intra-class correlation coefficient; LASSO, least absolute shrinkage and selection operator; ROC, receiver operating characteristic; SHAP, Shapley additive explanation.

Seven commonly used ML algorithms were used to construct predictive models for the training set: LR, SVM, KNN, RF, DT, NB, and NN. Model performance was evaluated using receiver operating characteristic (ROC) curves and the area under the ROC curve (AUC). The AUC values were compared between models using the DeLong test. Additionally, accuracy, precision, the F1 score, and recall were calculated to provide a comprehensive assessment of model performance. Model construction and evaluation were performed using Python version 3.8.0 (Python Software Foundation; Beaverton, OR, USA). **Figure 2** illustrates the workflow.

To mitigate potential selection bias and address class imbalance (EGFR−: EGFR+ ≈ 2:1), we applied stratified 10-fold cross-validation, ensuring class proportions were preserved in each fold. We also tested a soft-voting ensemble (RF + SVM + DT), which showed comparable performance to the best individual classifier (see **Supplementary Table S2**). Classifier hyperparameters are summarized in **Supplementary Table S3**.

### 2.6 Model interpretation with SHAP

The SHAP method, a game-theory-based method, provides valuable insights into the influence of individual features by quantifying their contributions to model predictions. This method provides both global and sample-level insights into model behavior. In this study, we applied the SHAP method to interpret the constructed ML models, addressing the “black-box” challenges commonly associated with these algorithms. All analyses were conducted using SHAP software (version 0.44.1). Feature importance plots and summary plots were generated, and representative cases were selected to create SHAP force plots, thereby enhancing our understanding of the model predictions.

### 2.7 Statistical analyses

All statistical analyses were conducted using R (version 4.3.3; https://www.r-project.org) and Python (version 3.8.0). Continuous variables are expressed as mean with standard deviation, whereas categorical variables are reported as frequency and percentage. The clinical characteristics of the EGFR+ and control groups were compared using *t*-tests for continuous variables and chi-square test (or Fisher’s exact test when appropriate) for categorical variables. Seven ML algorithms were employed to construct predictive models, and their performances were evaluated using ROC curves. The SHAP analysis was applied to investigate the contributions of different variables to risk prediction. Statistical significance was defined as P < 0.05 for all analyses.

## 3 Results

### 3.1 Clinicopathological data

In total, 321 patients with breast cancer were included in the study, of whom 111 (34.6%) had EGFR+ status and the remaining 210 (65.4%) had EGFR- status. There were no significant differences between the groups in terms of age, maximum tumor diameter, irregular shape, height-to-width ratio, presence of microcalcifications, posterior shadowing, or blood flow signals (**Table 1**). No statistically significant differences were found based on t-test for continuous variables and chi-square test for categorical variables.

**Table 1:** Comparison of clinical and ultrasound characteristics of the patients.

Characteristics	EGFR		X^2^/t	P-value
	Negative (n = 210)	Positive (n = 111)		
Age (year, X ± SD)	51.39 ± 12.38	49.71 ± 11.30	1.187	0.236
Maximum diameter (mm, X ± SD)	21.15 ± 9.14	20.10 ± 9.29	0.976	0.330
Irregular shape			2.328	0.127
Negative (n, %)	69, 21.5	46, 14.3		
Positive (n, %)	141, 43.9	65, 20.2		
Taller than wide			2.061	0.151
Negative (n, %)	168, 52.3	81, 25.2		
Positive (n, %)	42, 13.1	30, 9.3		
Microcalcification			2.748	0.097
Negative (n, %)	122, 38.0	75, 23.4		
Positive (n, %)	88, 27.4	36, 11.2		
Shadow			0.498	0.481
Negative (n, %)	137, 42.7	68, 21.2		
Positive (n, %)	73, 22.7	43, 13.4		
CDFI			1.329	0.249
Negative (n, %)	105, 32.7	63, 19.6		
Positive (n, %)	105, 32.7	48, 15.0		

EGFR, epidermal growth factor receptor; CDFI, color Doppler flow imaging. P-values were computed using the Mann-Whitney U test.

### 3.2 Feature selection

**Figure 3:**
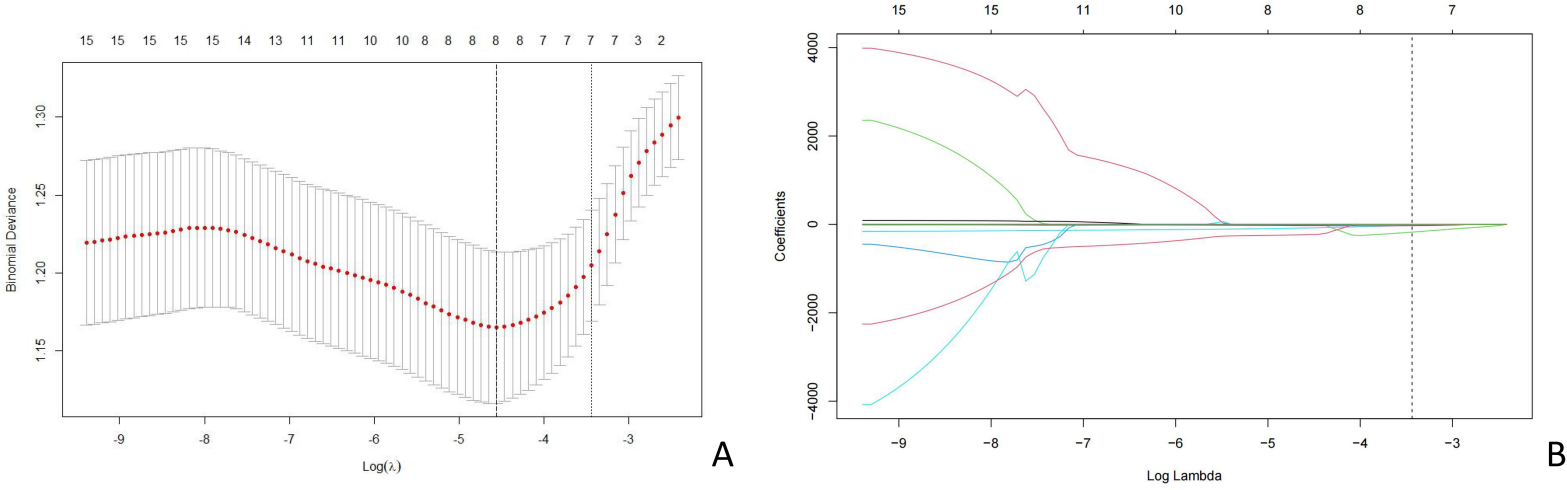
LASSO coefficient profiles of the risk factors. **(A)** Distribution of the coefficients of 16 features after LASSO regression. **(B)** Cross-validation curve used to determine the optimal regularization parameter (λ). LASSO, least absolute shrinkage and selection operator.

A total of 464 radiomic features were extracted from the breast cancer ultrasound images of each patient. Among these, 335 features exhibited inter- and intra-observer ICC values of >0.75, indicating good consistency and suitability for further analysis. After consistency testing, *t*-tests were conducted on these 335 features, resulting in the retention of 16 features. Finally, the LASSO regression method with 10-fold cross-validation was applied, yielding eight features for constructing the radiomics model (**Figure 3**). The Least Absolute Shrinkage and Selection Operator (LASSO) regression with 10-fold cross-validation was used to select the most informative features from the 16 features that passed the univariate test. **Figure 3A** displays the binomial deviance of the LASSO regression across log(λ) values, with the optimal value selected via 10-fold cross-validation. **Figure 3B** illustrates the coefficient profiles of features as a function of λ. Eight features with non-zero coefficients at the optimal λ were selected for model construction. This sensitivity to boundary placement has also been reported in phantom and repeatability studies, where GLCM and GLRLM features showed reduced robustness compared to first-order and shape features (22, 23).

### 3.3 Model performance

**Figure 4:**
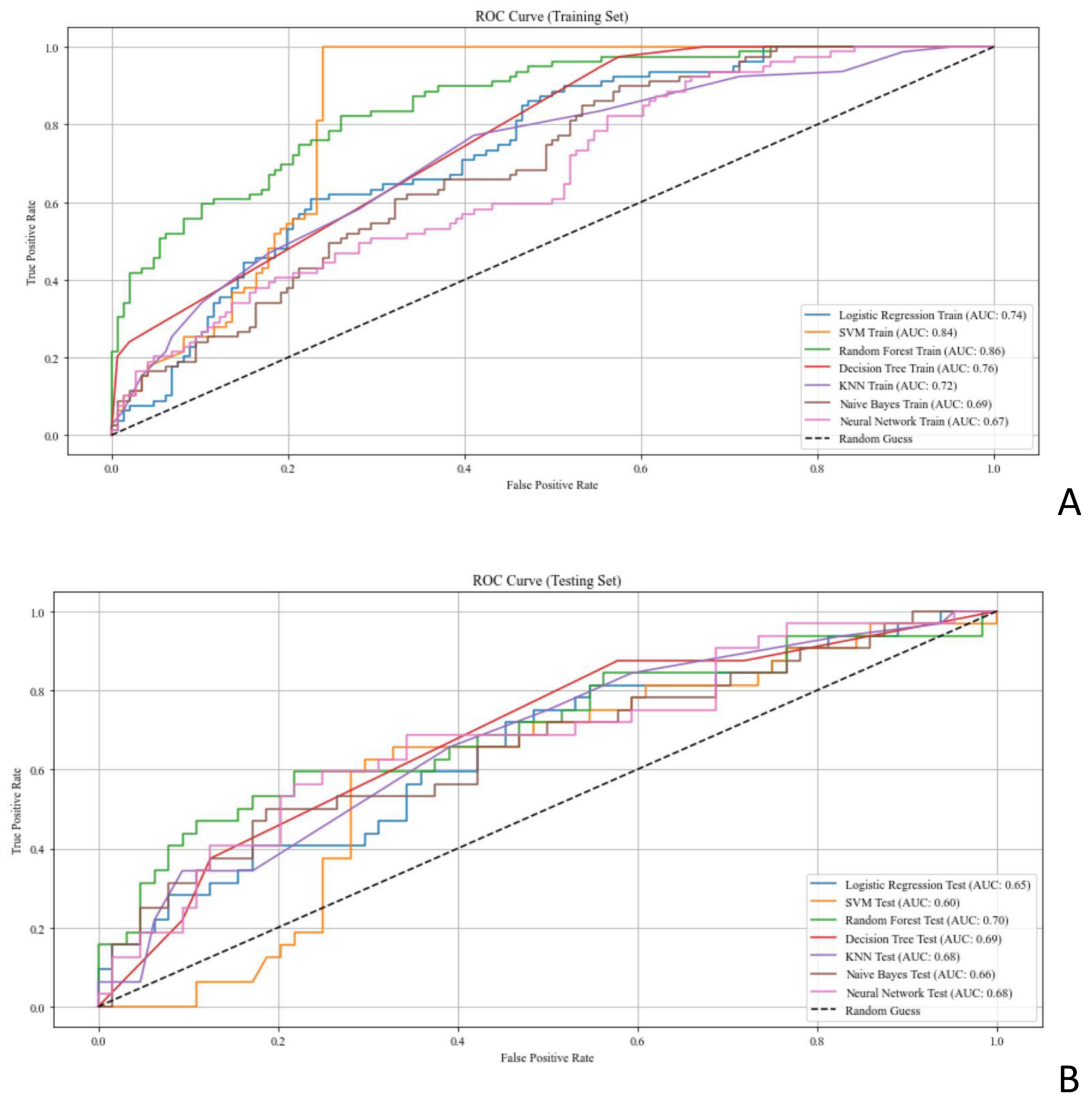
ROC curves assessing the performance of ML models for predicting EGFR status in patients with breast cancer. **(A, B)** ROC curves of the ML models in the **(A)** training set and **(B)** test set. AUC, area under the curve; EGFR, epidermal growth factor receptor; ML, machine learning; ROC, receiver operating characteristic.

The selected features were input into the seven ML models. The performance of these models was evaluated using ROC curves for both the training and test sets (**Figure 4**; **Table 2**). In the training set, the LR model achieved an AUC of 0.74. Delong’s test indicated that the RF model had the highest AUC, significantly outperforming the LR, SVM, DT, KNN, NB, and NN models (P < 0.001). In the test set, the RF model (AUC = 0.70) outperformed the SVM model (AUC = 0.60, P < 0.05). Radar plots were used to visualize the relative importance of selected features across different models. However, no significant differences were observed between the RF model and the LR, KNN, DT, NB, and NN models (P > 0.05). Although several between-model differences on the hold-out test were not statistically significant, cross-validation showed that RF delivered balanced performance with a higher mean F1-score (0.54 ± 0.12) on average, supporting its selection as the final model. Beyond the 7:3 hold-out test (RF AUC = 0.76; F1 = 0.58), stratified 10-fold cross-validation yielded consistent performance (AUC 0.82 ± 0.08; F1 0.54 ± 0.12), supporting model robustness (**Supplementary Table S4**).

**Table 2:** Comparison of the performance of machine learning models in training and test sets.

Sets	Models	AUC	Accuracy	Precision	F1-score	Recall
Training set	RF	0.86	0.76	0.63	0.81	0.71
	LR	0.74	0.60	0.47	0.91	0.62
	SVM	0.84	0.67	0.59	0.22	0.31
	DT	0.76	0.62	0.48	0.97	0.64
	KNN	0.72	0.68	0.65	0.22	0.32
	NB	0.69	0.59	0.45	0.78	0.57
	NN	0.67	0.68	0.72	0.16	0.27
Test set	RF	0.70	0.62	0.46	0.66	0.54
	LR	0.65	0.48	0.81	0.31	0.77
	SVM	0.60	0.58	0.25	0.12	0.17
	DT	0.69	0.53	0.41	0.88	0.55
	KNN	0.68	0.70	0.64	0.22	0.33
	NB	0.66	0.56	0.41	0.72	0.52
	NN	0.68	0.19	0.67	0.19	0.29

AUC, area under the curve; RF, random forest; LR, logistic regression; SVM, support vector machine; DT, decision tree; KNN, k-nearest neighbors; NB, naive Bayes; NN, neural network.

**Figure 5:**
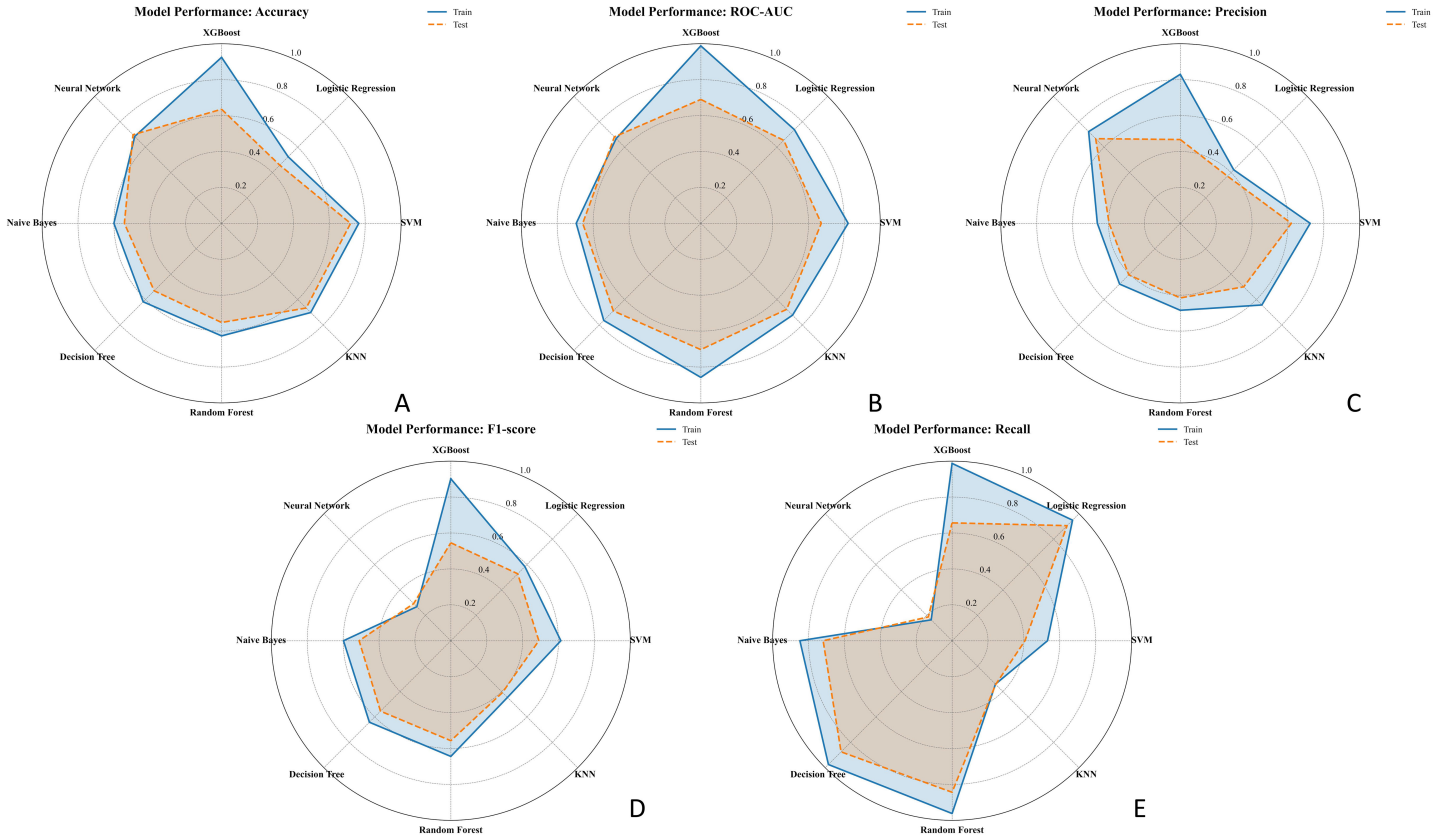
Radar charts comparing the performance of seven ML models in predicting EGFR status across five metrics: **(A)** Accuracy, **(B)** AUC, **(C)** Precision, **(D)** F1-score, and **(E)** Recall. Each chart displays the performance in both the training set (solid blue line) and test set (dashed orange line). Models compared include Logistic Regression, SVM, XGBoost, Random Forest, KNN, Decision Tree, and Neural Network.EGFR, epidermal growth factor receptor; ML, machine learning; AUC, area under the curve.

An exploratory soft-voting ensemble achieved performance comparable to RF (10-fold AUC 0.73 ± 0.10 vs. RF 0.82 ± 0.08; hold-out AUC both 0.76), suggesting limited incremental benefit on this dataset (**Supplementary Table S2**). The radar plots in **Figure 5** illustrate model performance metrics (AUC, accuracy, precision, recall, and F1-score) across classifiers, highlighting that Random Forest and XGBoost achieved the best overall generalization on both training and test sets.

### 3.4 Model interpretability

**Figure 6:**
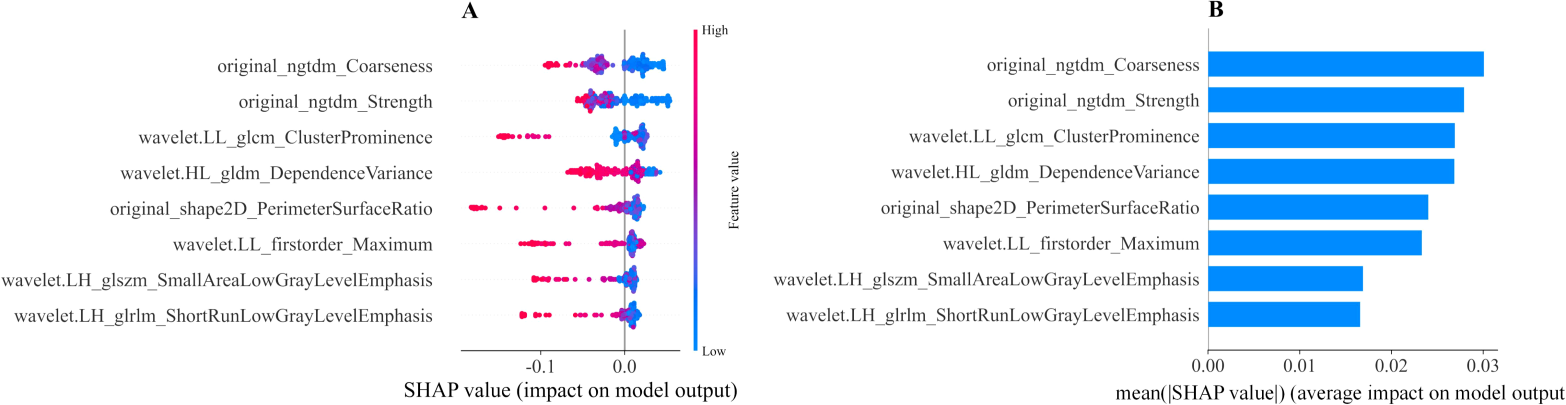
Interpretability of the ML radiomic model assessed using the SHAP method. **(A)** SHAP summary plot showing the impact of each feature on the model’s predictions. Individual dots represent patients, with different colors indicating varying levels of influence on the model’s output. **(B)** SHAP bar chart displaying the importance of each feature based on mean SHAP values. ML, machine learning; SHAP, Shapley additive explanation.

**Figure 7:**
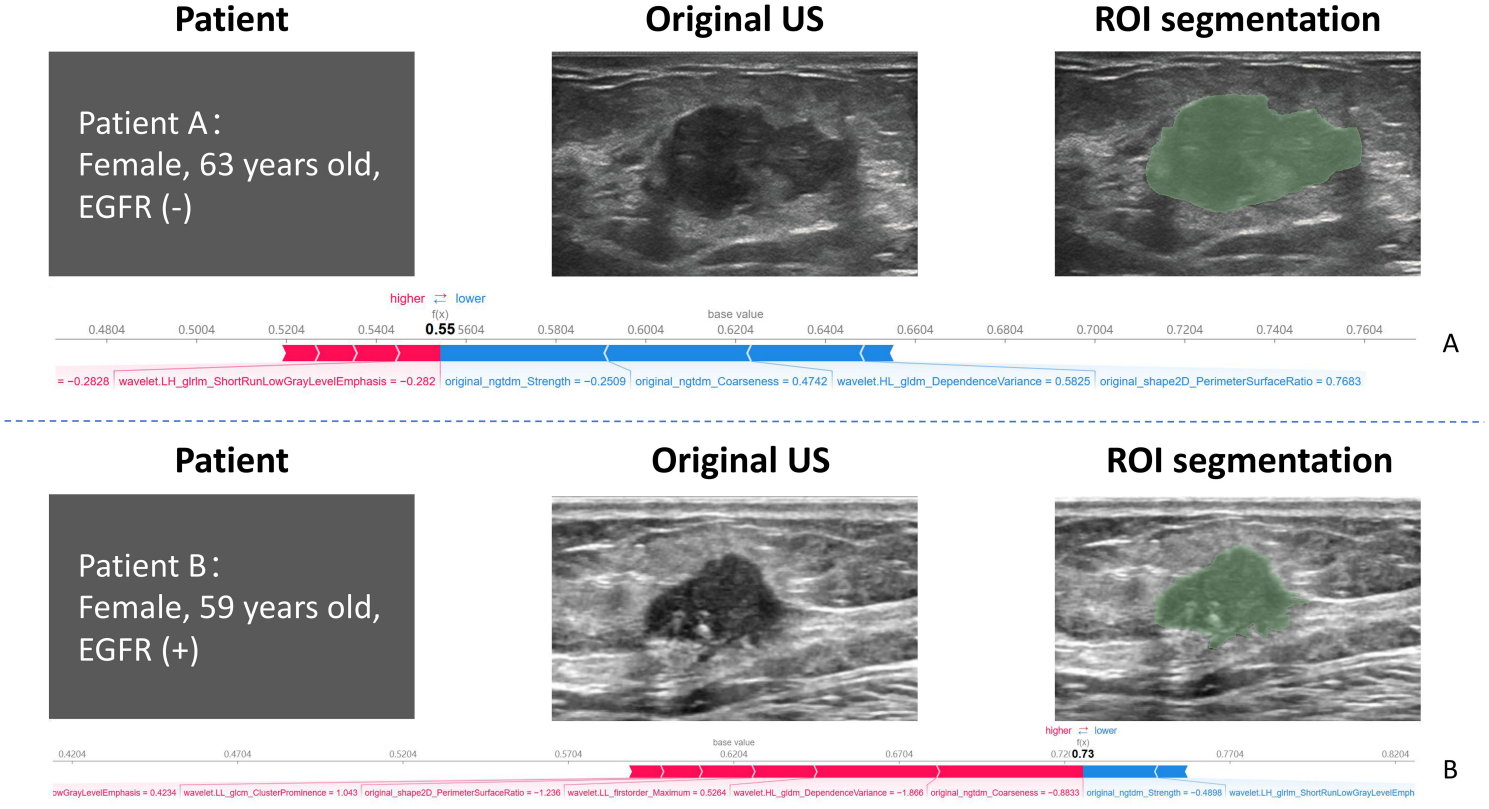
Representative examples of two patients with distinct EGFR expression status. Patient **(A)** (top row): 63-year-old female with EGFR-negative tumor. Patient **(B)** (bottom row): 59-year-old female with EGFR-positive tumor. For each case, the original grayscale ultrasound image, manual ROI segmentation, and SHAP summary output are shown. SHAP values highlight the most influential radiomic features contributing to the model’s prediction for each individual. EGFR, epidermal growth factor receptor; SHAP, Shapley additive explanation.

We calculated the SHAP values for each ultrasound radiomic feature in the RF model. The SHAP feature importance scatter plot (**Figure 6A**) illustrates the distribution of SHAP values for each feature, with each point representing the SHAP value of a sample and the color indicating the feature value (e.g., high or low). As shown in the plot, original_ngtdm_Coarseness and original_ngtdm_Strength exhibit the widest distribution of SHAP values, highlighting their significant influence on the prediction model. The gradient from blue to red reflects the magnitude of the feature values, with high values represented in red and low values in blue, emphasizing the nonlinear effect of these features on the prediction output. The SHAP feature importance bar chart (**Figure 6B**) ranks the features according to their absolute mean SHAP values, reflecting their relative importance in the model’s overall predictions. The top-ranked features, original_ngtdm_Coarseness and original_ngtdm_Strength, were identified as the primary drivers of the model’s predictions. Among the selected features, texture features such as original_ngtdm_Coarseness, original_ngtdm_Strength, and wavelet.LL_glcm_ClusterProminence, as well as 2D shape features like original_shape2D_PerimeterSurfaceRatio, demonstrated significant differences between EGFR+ and EGFR− tumors. Specifically, EGFR+ tumors exhibited lower values in original_ngtdm_Coarseness (0.00105 vs. 0.00153, p = 0.0012), original_ngtdm_Strength (5.06 vs. 7.55, p = 0.0015), and PerimeterSurfaceRatio (0.119 vs. 0.140, p = 0.0178), indicating finer texture and more compact tumor structures compared to EGFR− tumors (**Supplementary Table S5**). Other features, such as wavelet.LL_glcm_ClusterProminence and wavelet.HL_gldm_DependenceVariance, also contributed significantly, whereas lower-ranked features had smaller contributions. **Figure 7** presents two representative patients: one with EGFR-negative (Patient A) and one with EGFR-positive status (Patient B). For each case, the original grayscale ultrasound image, ROI segmentation, and SHAP output are shown. The SHAP visualizations illustrate how specific radiomic features influenced the model’s prediction at the individual level. Notably, texture-based descriptors such as original_ngtdm_Coarseness and original_ngtdm_Strength demonstrated substantial contributions, reflecting local intensity granularity and uniformity. These radiomic patterns, including lower coarseness and strength, indicate finer and more homogeneous texture in EGFR+ tumors. This observation is consistent with the hypothesis that EGFR-overexpressing tumors may exhibit higher cellular density and less architectural heterogeneity, as also suggested in previous studies (24, 25).

## 4 Discussion

In this study, we developed and validated an interpretable ML model using ultrasound radiomic features to predict EGFR expression status in breast cancer. The random forest (RF) model achieved the highest performance among seven machine learning models, with an AUC of 0.86 on the training set and 0.70 on the hold-out test set. Furthermore, 10-fold stratified cross-validation confirmed the robustness of the RF model (AUC = 0.82 ± 0.08; F1-score = 0.54 ± 0.12), supporting its selection as the final model. Although the test set AUC was moderate (~0.76), the RF model consistently outperformed others in recall and F1-score, metrics that are crucial for clinical risk stratification. These results are in line with previous radiomics studies reporting similar performance for EGFR prediction in other cancers (26, 27).

The novelty of this study lies in the integration of ultrasound radiomics and ML techniques to develop a high-performance RF model that demonstrates superior performance across multiple evaluation metrics. This model provides valuable technical insights for advancing the development of clinical diagnostic systems. Prior studies have explored EGFR prediction primarily in non-small cell lung cancer using PET/CT or multiparametric MRI, achieving AUCs ranging from 0.61 to 0.85. In contrast, our model achieved comparable or superior performance (AUC = 0.76–0.82) using cost-effective, non-invasive ultrasound imaging (26–28). This approach may offer a practical alternative for wider clinical application, particularly in settings lacking advanced imaging modalities.

In this study, we integrated ultrasound imaging with ML, validating the potential of ultrasound radiomics in quantifying tumor heterogeneity. These findings align with those of previous studies that successfully predicted ER, progesterone receptor, HER2, and Ki-67 expression statuses in breast cancer using radiomic analysis (29–31). Notably, by predicting the EGFR expression status, this study expands the application of radiomics to the molecular subtyping of breast cancer. Eight key radiomic features were selected to construct the ultrasound radiomics model: two NGTDM features, one gray-level dependence matrix feature, one GLRLM feature, one GLSZM feature, one GLCM feature, one shape feature, and one first-order statistical feature. These features included six texture features, one shape feature, and one first-order statistical feature. The six texture features capture the complexity of tumor texture, which is critical to identifying and classifying spatial heterogeneity within tumor lesions (32, 33). This finding underscores the importance of texture features in predicting high EGFR expression. Additionally, the RF model developed in this study provides a comprehensive analysis of tumor characteristics by integrating texture, shape, and first-order statistical features, thereby enhancing the accuracy and reliability of tumor predictions. By combining these diverse feature types, the RF model captures tumor image information more comprehensively, leading to more precise predictions and diagnoses. This integrated analysis offers new perspectives and methodologies for diagnosing and predicting EGFR mutations in patients with breast cancer, demonstrating potential for clinical application.

The SHAP values were applied to the RF model to enhance both predictive performance and interpretability. With these values, we can evaluate the contribution of each feature to the model’s output by analyzing all possible feature combinations, providing consistent and locally accurate attribute values for each feature. The SHAP analysis of the RF model revealed that original_ngtdm_Coarseness and original_ngtdm_Strength had the most significant effect on EGFR expression status prediction. These features quantify subtle variations in tumor texture, which aligns with the recognized importance of texture features in tumor classification and prediction in the field of radiomics (34, 35). Using the SHAP method, we quantified the importance of features and revealed their nonlinear effects on the model’s decision-making process, thereby enhancing its transparency and clinical credibility. Applying these insights to the RF model enables users to better understand its predictions and the rationale behind its decisions. The detailed insights and explanations of risk factors presented in the results provide clinicians with a more informed perspective, fostering evidence-based decision-making rather than blind reliance on algorithm outputs. Moreover, individualized explanations help clinicians understand why the model suggests specific decisions for high-risk cases, supporting personalized patient management.

Several limitations merit consideration. First, this was a single-center, retrospective study with a modest sample size and a class imbalance (EGFR–:EGFR+ ≈ 2:1), which may limit generalizability. Although we applied stratified sampling, class weighting, and 10-fold cross-validation to minimize bias, external validation across multiple institutions is necessary. Second, the manual segmentation of ROIs introduces subjectivity and may impact reproducibility; future work should explore automated deep learning-based segmentation. Lastly, although ensemble learning was explored, it did not outperform the RF model, potentially due to data scale and signal-to-noise characteristics.

## 5 Conclusions

In this study, we developed an interpretable ML model based on ultrasound radiomic features to predict the EGFR expression status in breast cancer. The model demonstrated excellent predictive performance, which was further enhanced using the SHAP method. The SHAP values improved both global and local interpretability, providing reliable support for precise and non-invasive diagnosis. Ultrasound radiomics offers a more cost-effective and non-invasive alternative to invasive testing methods, making it particularly suitable for patients who are unable to undergo such procedures. This approach shows clinical potential for widespread applications in breast cancer diagnosis and management. Among the top-ranked SHAP features, original_ngtdm_Coarseness, original_ngtdm_Strength, and wavelet.LL_glcm_ClusterProminence not only exhibited significant intergroup differences between EGFR+ and EGFR− tumors but also reflected texture compactness and heterogeneity, suggesting a strong association with the underlying biological mechanisms of EGFR overexpression.

## Data Availability

The original contributions presented in the study are included in the article/**Supplementary Material**. Further inquiries can be directed to the corresponding authors.
